# Eugenol—From the Remote Maluku Islands to the International Market Place: A Review of a Remarkable and Versatile Molecule

**DOI:** 10.3390/molecules17066953

**Published:** 2012-06-06

**Authors:** Guy P. Kamatou, Ilze Vermaak, Alvaro M. Viljoen

**Affiliations:** Department of Pharmaceutical Sciences, Faculty of Science, Tshwane University of Technology, Private Bag X680, Pretoria 0001, South Africa; Email: kamatougp@tut.ac.za (G.P.K.); vermaaki@tut.ac.za (I.V.)

**Keywords:** clove oil, *Eugenia caryophyllata*, eugenol, mechanism of action, pharmacological activity, *Syzygium aromaticum*, toxicity

## Abstract

Eugenol is a major volatile constituent of clove essential oil obtained through hydrodistillation of mainly *Eugenia caryophyllata* (=*Syzygium aromaticum*) buds and leaves. It is a remarkably versatile molecule incorporated as a functional ingredient in numerous products and has found application in the pharmaceutical, agricultural, fragrance, flavour, cosmetic and various other industries. Its vast range of pharmacological activities has been well-researched and includes antimicrobial, anti-inflammatory, analgesic, anti-oxidant and anticancer activities, amongst others. In addition, it is widely used in agricultural applications to protect foods from micro-organisms during storage, which might have an effect on human health, and as a pesticide and fumigant. As a functional ingredient, it is included in many dental preparations and it has also been shown to enhance skin permeation of various drugs. Eugenol is considered safe as a food additive but due to the wide range of different applications, extensive use and availability of clove oil, it is pertinent to discuss the general toxicity with special reference to contact dermatitis. This review summarises the pharmacological, agricultural and other applications of eugenol with specific emphasis on mechanism of action as well as toxicity data.

## 1. Introduction

**Figure 1:**
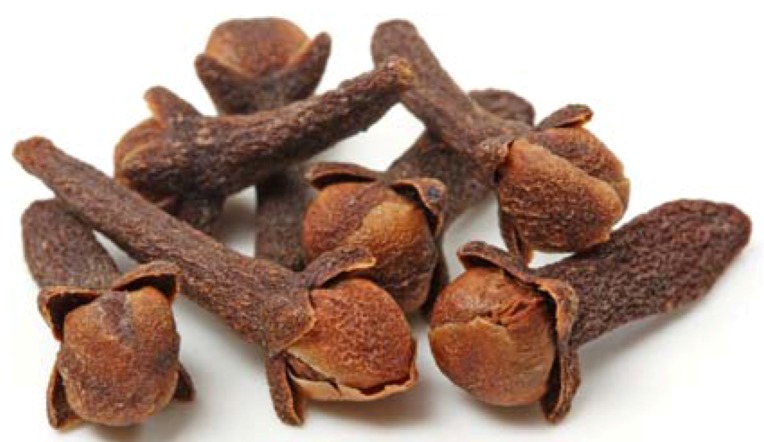
The dried flowerbuds of *Eugenia caryophyllata*, known as cloves.

The spice known as clove (Figure 1) is the dried flowerbud of the clove tree, *Eugenia caryophyllata* Thunb. (=*Syzygium aromaticum *(L.) Merr. & L.M. Perry) (Myrtaceae), which has a nail-like appearance leading to its vernacular names in several languages such as Dutch (nagel), Spanish (clavo) and Portugese (cravo). Clove, native to the small islands of Maluku in Eastern Indonesia also known as the “Spice Islands”, has been traded from one end of the World to the other, being a highly sought after commodity in medieval Europe for medicinal and culinary purposes. During the fourteenth century the clove trade acted as a stimulant in the establishment of commerce at ports especially in Asia and Europe where it was traded for large profits. The high clove trade price inspired exploration expeditions in the search for new sources of this highly praised spice and the establishment of new sea routes. Throughout the following centuries its trade went through several phases such as increased trade prices, struggle over control of the industry, warfare, decreased trade prices and even smuggling of seedlings for cultivation [1,2,3].

Clove has been used in ancient China as a spice and fragrance for more than 2,000 years. Medicinally, the well-known traditional remedy of applying clove oil to treat a toothache was documented for the first time in 1640 in ‘Practice of Physic’ in France [4,5]. In Chinese traditional medicine clove oil has been used as carminative, antispasmodic, antibacterial and antiparasitic agent, while the buds were used to treat dyspepsia, acute/chronic gastritis and diarrhoea [5,6]. Several scientific studies have been carried out on *E. caryophyllata *oil and its main volatile constituent eugenol, revealing pharmacological properties such as anaesthetic and analgesic effects. In addition, antimicrobial, anti-oxidant, anti-inflammatory, anticonvulsant [6], anticarcinogenic [1], antimutagenic [7], repellant and antifumigant activities [8] have been reported.

**Figure 2:**
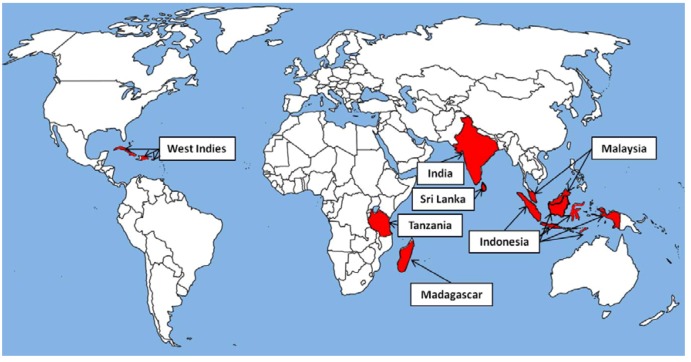
The major clove producers of the world are the West Indies, Madagascar, Tanzania, India, Sri Lanka, Indonesia and Malaysia.

Indonesia, India, Malaysia and Sri Lanka are the major Asian producers of clove but greater quantities are produced in the West Indies, Madagascar and Tanzania, especially Zanzibar (Figure 2). The harvesting of the unopened flower buds is done by hand-picking and therefore labour-intensive and the dry weight of the buds is only one third of the original weight [3]. In 2001 it was estimated that approximately 2,000 tonnes of oil are produced annually and 2006 figures estimated a gross market value of US$30–70 million per annum through its function as flavour and fragrance ingredient and antibacterial agent [9]. The name of the main constituent of clove oil, eugenol, is derived from the species name *Eugenia caryophyllata* which contains a high level of eugenol (45–90%) in addition to acetyleugenol, chavicol, acetyl salicylate and humulenes [1]. Eugenol was first isolated in 1929 and commercial production commenced in the United States in the 1940s [5]. Eugenol can be produced synthetically, the most practical method being the allylation of guaiacol with allyl chloride. However, eugenol is predominantly prepared from natural oil sources by mixing the essential oil with an excess of aqueous sodium (3%) or potassium hydroxide solution and shaking, leading to the formation of a phenolic alkali salt. The insoluble non-phenolic portion is then extracted with a solvent or via steam distillation. The undissolved portion is removed, the alkali solution acidified at low temperatures and the liberated eugenol purified by fractional distillation [10].

Eugenol is an extraordinarily versatile molecule and has been included as an ingredient in cosmetics and several popular perfumes, for example Opium^®^ and Kouros^®^ by Yves Saint Laurent [11], as a spicy flavourant in whisky [12], ice cream, baked goods and candy in restricted concentrations [9], mouthwashes, pharmaceutical and dental preparations [5,13]. In dentistry it is used in combination with zinc oxide to form a polymerised eugenol cement used for surgical dressings, temporary fillings, pulp capping agents and cavity liners [9]. Eugenol is the key ingredient in clove cigarettes unique to Indonesia, known as kretek. These cigarettes were extremely popular and about 10 billion were produced in 1939, 20 billion in 1972 and 67 billion in 1982, but their use has declined sharply due to reports of deleterious health effects. Eugenol was the first natural compound used in the synthesis of vanillin during the late 19th and early 20th century. However, most vanillin nowadays is produced from phenol or lignin. Eugenol is also used as an industrial source to produce isoeugenol and methyleugenol [5,9,14].

In recent years, eugenol has attracted the attention of many researchers because of its anti-inflammatory and chemopreventive effects, as well as its superior anti-oxidant activity due the presence of its phenolic group [15]. As a result of its broad range of pharmacological and biological activities, studies on eugenol and clove products still remains a research priority. It is therefore of significant value to coherently unite some of the most noteworthy research findings related to eugenol to highlight its importance in phytotherapy and industry as well as to elucidate its mechanisms of action where possible.

## 2. Natural Sources of Eugenol

Eugenol has been identified in several aromatic plants such as *Myristica fragrans* Houtt. (nutmeg), *Cinnamomum* *verum* J.Presl (true cinnamon), *C. loureirii* Nees. (Saigon cinnamon), *Ocimum gratissimum* Forssk. (basil) and *Ocimum basilicum* L. (sweet basil). However, *Eugenia caryophyllata* (=*Syzygium aromaticum*) can be considered the principal natural source of this compound as it represents between 45 and 90% of the total oil. Commercial eugenol is derived from clove bud/leaf oil, cinnamon leaf oil or basil obtained through steam distillation which is then further refined [1,5,10]. 

## 3. Chemical and Physical Properties

**Figure 3:**
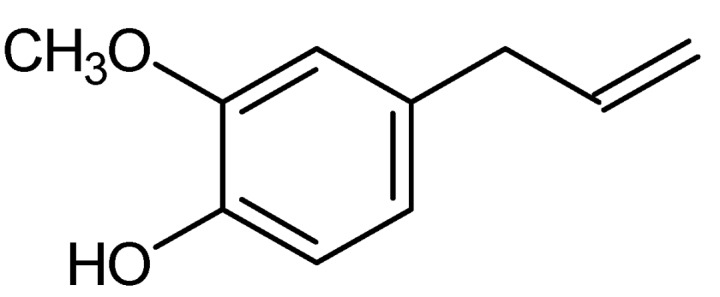
Chemical structure of eugenol.

Eugenol (C_10_H_12_O_2_), a phenylpropanoid, is an allyl chain-substituted guaiacol (Figure 3), which is weakly acidic, slightly soluble in water and soluble in organic solvents. It is a clear to pale yellow liquid with a characteristic and pleasant odour of cloves and a spicy pungent taste. Large quantities of eugenol are used in detergents and soaps for their spicy aroma but they cause discolouration due to their phenol structure. The ester form is non-discolouring and stable but is not often used in applications such as perfumes as they do not possess the powerful spicy notes of the free phenol compound [5,10]. 

## 4. Pharmacological Properties of Eugenol and Possible Mechanisms of Action

Several* in vitro* and* in vivo* studies have been conducted to determine the pharmacological properties ascribed to eugenol and to elucidate mechanism of action. 

### 4.1. Anti-Infective Activity

#### 4.1.1. Antibacterial Activity

For centuries, natural products have been used to treat microbial infections and numerous essential oil molecules have demonstrated the ability to inhibit the growth of various pathogens. Singh *et al.* [16] investigated the effect of eugenol on the growth of Gram-positive (*Bacillus cereus*; *B. subtilis*; *Staphylococcus aureus*) and Gram-negative (*Escherichia coli*; *Salmonella typhi*; *Pseudomonas aeruginosa*) bacteria using the agar well diffusion method. At 1,000 ppm, eugenol inhibited the growth of the bacteria and complete inhibition was obtained against *P. aeruginosa* at a high concentration of 2,000 ppm. This inhibition was high in comparison to ampicillin (1 mg/mL) used as a positive control. Several other studies have confirmed the antibacterial activity of eugenol against various pathogens such as *E. coli*, *B. cereus*, *Helicobacter pylori*, *S. aureus*, *S. epidermidis*, *Streptococcus pneumoniae* and *S. pyogenes* amongst numerous others [17,18,19,20]. An investigation was conducted to evaluate the effect of eugenol on adherence and biofilms of two pathogenic *P. aeruginosa* isolates. The treatment of *P. aeruginosa* with eugenol (0.5%) resulted in 60% adherence inhibition for *P. aeruginosa* (CIP A22) and 69% for *P. aeruginosa* (ATCC27853). Inhibition of more than 90% was obtained when eugenol was tested against *P. aeruginosa* (ATCC 27853) biofilms [21]. Combination therapy is often needed in the treatment of serious infection and to reduce the risk of resistant strain development. In traditional medicine, healers mostly rely on a combination of plants to treat diseases [22]. Several studies have investigated the combination of an essential oil molecule with a conventional antibiotic to determine possible synergistic interaction. For instance, the combination of eugenol with vancomycin and β-lactam antibiotics resulted in an increase of activity by a factor of 5–1,000 with respect to their individual MIC values. This synergistic effect could be explained by the fact that eugenol is able to damage the membrane of Gram-negative bacteria. It was found that a concentration of 1 mM damaged nearly 50% of the bacterial membrane allowing increased penetration of vancomycin and β-lactam antibiotics and therefore a greater antimicrobial effect [23]. The synergistic interaction of eugenol with ampicillin and gentamicin was investigated using time-kill studies. After 60 min of treatment, the rate of killing in units of bacteria evaluated by CFU/mL was higher when eugenol was combined with either antibiotic than when tested alone, suggesting a synergistic interaction [24]. In another investigation, the antibacterial activity of eugenol, cinnamaldehyde, thymol, carvacrol and their combinations were investigated against the Gram-negative bacteria *E. coli* using the broth micro-dilution assay. Eugenol possessed the lowest antibacterial activity (MIC value: 1,600 mg/L) in comparison with the other three compounds which had an MIC value of 400 mg/L. However, when eugenol was combined with cinnamaldehyde, thymol and carvacrol, synergistic interactions were noted resulting in MIC values of 400, 100, 100 mg/L, respectively [25]. 

The biological activity of essential oils is generally investigated without emphasis on the mechanism of action. The mechanism of action of eugenol was studied to evaluate the effect on the bacterial membranes of *Listeria monocytogenes*, *Streptococcus pyogenes*, *Proteus vulgaris* and *E. coli* by observing changes in membrane composition and monitoring the leakage of protein and lipid. The study revealed that eugenol induced cell lysis through leakage of protein and lipid contents. In addition both the cell wall and membrane of the treated Gram-negative and Gram-positive bacteria were significantly damaged and eugenol caused high protein content leakage after 120 min of exposure [26]. 

The results from tumour necrosis factor (TNF) release and haemolysin assays indicated that *S. aureus* cultured together with graded subinhibitory concentrations of eugenol (16 to 128 μg/mL) dose-dependently decreased the necrosis factor-inducing and haemolytic activities of culture supernatants and significantly reduced the production of staphylococcal enterotoxin A. Therefore, eugenol could be applied to food products as a novel food antimicrobial agent both to inhibit the growth of bacteria and to suppress the production of exotoxins by *S. aureus* [27]. The combination of cinnamate and eugenol as preservative may also be useful to protect food products. These compounds in an optimal combination of 1.35 mM cinnamate and 0.12 mM eugenol produced a bactericidal synergistic effect against *E. coli* O157:H7, *Salmonella typhi* and *L. monocytogenes* and an additive effect against *S. aureus*. In addition, the results showed that these micro-organisms expressed efflux pumps in response to antimicrobial activity [28].

#### 4.1.2. Antifungal Activity

Candidiasis therapy is becoming problematic due to the development of antimicrobial resistance to certain drugs. Therefore, combination therapy as an alternative treatment method becomes more important. The *in vitro* antifungal activity of eugenol and methyleugenol singularly and in combination with fluconazole (antifungal drug) against 64 fluconazole-sensitive and 34 fluconazole-resistant clinical *C. albicans* strains was studied. All the strains tested were susceptible and the combination of eugenol/methyleugenol with fluconazole resulted in a synergistic interaction. These results suggest that a combination of eugenol/methyleugenol with fluconazole or amphotericin B could be an alternative method to treat fluconazole-resistant or multi-drug resistant isolatesagainst of *C. albicans* [29,30]. 

Eugenol was evaluated *in vivo *for the prophylaxis and treatment of experimental vaginal candidiasis on immunosuppressed rats. The results indicated that prophylactic eugenol treatment after 10 days of inoculation reduced the number of colonies of *C. albicans* in the vaginas of infected rats by 98.9% [31]. The *in vivo* antifungal activity of eugenol-loaded lipid nanoparticles was evaluated using a model of oral candidiasis in immunosuppressed rats. Significant improvement was obtained for all the groups by the 8th day with the log CFU (colony forming units) value for the eugenol solution slightly lower (3.39 ± 0.08) than for the control group treated with saline (3.89 ± 0.03) [32]. In another study, the anticandidal activity of eugenol in terms of MIC was 0.5 mg/mL. The ability of eugenol to interfere with the architecture of the envelope of *C. albicans* was further investigated. Exposure to eugenol (0.5 mg/mL) was found to induce a dramatic change in the envelope morphology. This interferes with adhesiveness and transition to the hyphal form thereby reducing the ability of *C. albicans* to colonise the host tissues [33]. The time-kill method was used to assess the inhibitory effect of eugenol and other terpenoids. Eugenol was highly toxic to *C. albicans*, killing 99.9% inoculum within seven minutes of exposure. It was shown that this compound acts by affecting membrane integrity and causes arrest of the cell cycle [34].

The effect of eugenol was evaluated against 38 *Candida* species isolated from denture-wearers and 10 collection *Candida* strains using the CLSI M27-A3 broth microdilution method. It was found that eugenol exhibited antifungal activity with the MIC value ranging from 0.06 to 0.25% (v/v), while the minimum concentration of drug that inhibited 50% of the isolates tested (defined as MIC_50_) ranged from 0.06 to 0.12% (v/v) [35]. MIC and skin lesion scoring tests were performed *in vivo* to evaluate the antifungal effect of eugenol in a guinea pig model infected by *Microsporum gypseum*. Eugenol exhibited promising activity with the MIC value ranging from 0.01 to 0.03%. The skin lesion scoring test showed that eugenol was clinically effective at improving the lesion during the first week of application. However, eugenol did not improve the skin lesions infected by *M. gypseum* as determined in the hair culture test [36]. Eugenol, like carvacrol and thymol, is lipophilic and can easily disperse between the fatty acyl chains making up the bilayers of cell membranes. Thus, it modifies the fluidity and permeability of cell membranes, thereby disturbing cell growth and envelope morphogenesis [37,38]. Ahmad *et al.* [29] observed that adding a methyl group to eugenol increases its antifungal activity.

Mycotoxigenic fungi cause plant diseases during storage and transport which may have an effect on human health. The essential oil components investigated, including eugenol, showed toxic effects on the *in vitro* mycelium growth against several *Penicillium*, *Fusarium *and *Aspergillus* species and *Alternaria alternata*. Several other studies have confirmed the antifungal activity of eugenol against pathogens such as *A. ochraceus*, *F. graminearum*, *F. moniliforme*, *Penicillium citrinum*, *P. viridicatum*, *Tricophyton rubrum*, *T. mentagrophytes*, *C. tropicalis* and *C. krusei* [16,39]. The antimycotic activities of eugenol was investigated with 10% eugenol dissolved in dimethyl sulfoxide (DMSO) using the microdilution method. Eugenol exhibited promising activity with MIC values of 0.5, 0.25 and 0.13 mg/mL against *Saprolegnia* spp., *A. klebsiana* and *A. piscicida*, respectively, and fungicidal concentrations of 1.0, 0.5 and 0.25 mg/mL [40]. Eugenol was also tested against *T. mentagrophytes* using the agar dilution method. At 0.15 mg/mL, eugenol completely inhibited the hyphal growth. It was also noted that at doses of 0.2 mg/mL of eugenol, the hyphae was distorted and collapsed [41]. Eugenol and other essential oil constituents (e.g. thymol, cinnamaldehyde, limonene) were tested against foodborne microflora of fresh-cut salad using the plate-count and Biolog methods. Promising results were obtained with eugenol inhibiting salad bacterial components at a concentration of 3 μL/mL [42]. 

The antifungal activity of *Cymbopogon flexuosus* Stapf essential oil and some individual oil components (citral, geraniol, eugenol, α-pinene and linalool) was evaluated by examining fungal growth and aflatoxin production of a toxigenic strain of *Aspergillus flavus*. The single compounds exhibited superior activity compared to the crude *Cymbopogon* essential oil. Eugenol exhibited the most potent antifungal and anti-aflatoxigenic activity with MIC values of 0.3 and 0.1 μL/mL, respectively. The activity of eugenol was even more potent than some known synthetic antimicrobials and exhibited a broad fungitoxic spectrum against *Aspergillus *species,* Alternaria alternata*, *Botryodiplodia theobromae*, *Cladosporium herbarum *and *Colletotrichum gloeosporioides* [43].

#### 4.1.3. Antiplasmodial Activity

Twenty essential oil constituents were evaluated for their antimalarial activity against the chloroquine-resistant strain *Plasmodium falciparum* (FCR-3). Eugenol exhibited some activity with an IC_50_ value of 753 μM which was lower than for other constituents such as nerolidol, linalyl acetae, α-pinene and pulegone (IC_50_ values ranging from 0.9 to 1.4 μM) [19].

#### 4.1.4. Antiviral Activity

The *in vitro* antiviral activity of eugenol has been tested against the herpes simplex-1 (HSV-1) and HSV-2 viruses. The replication of these viruses was inhibited with IC_50_ values of 25.6 μg/mL and 16.2 μg/mL against HSV-1 and HSV-2, respectively. Additional investigations revealed synergistic interactions with a combination of eugenol and acyclovir, a known antiviral drug. Studies have shown that application of eugenol delayed the development of herpes virus-induced keratitis in a mouse model [44]. Eugenol was evaluated for its anti-HSV properties on standard HSV-1(F), standard HSV-2(G) and ten HSV isolates using the plaque reduction assay. Only HSV-1 isolates 1 and 30 were inhibited by eugenol and the inhibition against these isolates was greater than for the extract obtained from the flower buds of *E. caryophyllata* [45]. 

#### 4.1.5. Anthelmintic Activity

The anthelmintic activity of the essential oil of *Ocimum sanctum *and eugenol was tested *in vitro* against *Caenorhabditis elegans*, which is a small, free living nematode. Eugenol exhibited promising activity with an ED_50_ value of 62.1 μg/mL and was suggested as the anthelmintic principle of *Ocimum sanctum *oil as it is the major component [46]. It is however important to note that one should be careful not to directly correlate the activity of an essential oil to its major constituents because minor constituents could also play an important role in pharmacological activity. 

The leishmanicidal activity of the essential oil of *Ocimum gratissimum*, rich is eugenol, and eugenol alone was investigated. The IC_50_ value of the essential oil against promastigotes and amastigotes was 135 and 100 μg/mL, respectively, and the activity of eugenol was more pronounced at an IC_50_ value of 80 μg/mL for promastigote forms. Morphological alterations were observed in *L. amazonensis* parasites after treatment with *Ocimum gratissimum* essential oil (rich in eugenol). Mitochondrial alterations occurred at the ultrastructural level, such as remarkable swelling, disorganisation of the inner membrane and increase in the number of cristae [47].

### 4.2. Anti-Inflammatory Activity

Inflammation and disease conditions are linked through the production of inflammatory mediators by macrophages and neutrophils. Inflammation results in increased expression and enzyme activity of cyclooxygenases (COX) 1 and 2. COX-2 in turn produces inflammatory mediators such as prostaglandin E_2_ (PGE_2_). These enzymes are involved in the inflammation and carcinogenesis processes, therefore it is recognised that potential COX-2 inhibitors can be considered anti-inflammatory or cancer chemopreventive agents. *Eugenia caryophyllata,* which contains eugenol and acetyleugenol as major constituents, was evaluated for potential anti-inflammatory action on COX-2 and 15-LOX enzymes. The oil exhibited strong inhibitory activity against COX-2 (58.15%) and 15-LOX (86.15%) enzymes at 10 μg/mL and 25 μg/mL, respectively [48]. The anti-inflammatory activity of eugenol was evaluated by inflammatory exudate volume and leukocyte migration in carrageenan-induced pleurisy and carrageenan-induced paw oedema tests in rats. Eugenol (200 and 400 mg/kg) was found to reduce the volume of pleural exudates without changing the total blood leukocyte count indicating the anti-inflammatory potential of eugenol [49]. 

Treatment of RAW264.7 cells with various concentrations of eugenol and activation with endotoxin lipopolysaccharide (LPS) showed that PGE_2_ production was significantly inhibited in a dose-dependent manner with an IC_50_ value of 0.37 μM. Nearly complete inhibition of PGE_2_ production was achieved at a eugenol concentration of 50 μM [50]. Co-treatment of RAW264.7 cells with LPS and eugenol (2.0–50 μM) caused concentration-dependent suppression of LPS-stimulated induction of COX-2 protein, indicating the specific inhibition of COX-2 protein expression by eugenol. In addition, eugenol was found to directly inhibit COX-2 enzyme activity (IC_50_ value: 2.7 μM) in intact cells [50]. The immunomodulatory/anti-inflammatory effects of eugenol (5, 10, 25, 50, 100 µg/well) was tested before and after incubation with LPS and the results revealed the inhibition of LPS action possibly through suppression of the NF-ΚB pathway. Eugenol (50, 100 µg/mL) inhibited interleukin (IL)-6 production either before or after addition of LPS and significantly counteracted LPS action when added after incubation [51]. In a previous study, Thompson and Eling [52] demonstrated the inhibition of manganese prostaglandin H synthase (Mn-PHS) activity in a cell-free enzyme system by eugenol. Although the mechanism of action could not be elucidated, it was suggested the competitive inhibition with the substrate arachidonic acid for the active site of PHS [52]. Other studies also speculated that the inhibitory effect of eugenol on PHS could be associated with the prevention of tyrosyl radical formation during the oxidation of arachidonic acid by PHS through a direct reaction with preformed tyrosyl radicals [53].

Treatment of RAW264.7 cells with various concentrations of eugenol and activation with endotoxin lipopolysaccharide (LPS) showed that PGE_2_ production was significantly inhibited in a dose-dependent manner with an IC_50_ value of 0.37 μM. Nearly complete inhibition of PGE_2_ production was achieved at a eugenol concentration of 50 μM [50]. Co-treatment of RAW264.7 cells with LPS and eugenol (2.0–50 μM) caused concentration-dependent suppression of LPS-stimulated induction of COX-2 protein, indicating the specific inhibition of COX-2 protein expression by eugenol. In addition, eugenol was found to directly inhibit COX-2 enzyme activity (IC_50_ value: 2.7 μM) in intact cells [50]. The immunomodulatory/anti-inflammatory effects of eugenol (5, 10, 25, 50, 100 µg/well) was tested before and after incubation with LPS and the results revealed the inhibition of LPS action possibly through suppression of the NF-ΚB pathway. Eugenol (50, 100 µg/mL) inhibited interleukin (IL)-6 production either before or after addition of LPS and significantly counteracted LPS action when added after incubation [51]. In a previous study, Thompson and Eling [52] demonstrated the inhibition of manganese prostaglandin H synthase (Mn-PHS) activity in a cell-free enzyme system by eugenol. Although the mechanism of action could not be elucidated, it was suggested the competitive inhibition with the substrate arachidonic acid for the active site of PHS [52]. Other studies also speculated that the inhibitory effect of eugenol on PHS could be associated with the prevention of tyrosyl radical formation during the oxidation of arachidonic acid by PHS through a direct reaction with preformed tyrosyl radicals [53].

5-Lipoxygenase (5-LOX) is the key enzyme in the biosynthetic pathway of leukotrienes. Leukotrienes are implicated in the pathophysiology of inflammatory disorders like asthma and allergic rhinitis [54]. Raghavenra *et al.* [54] found that eugenol inhibited the 5-LOX enzyme in human polymorphonuclear leukocytes with an IC_50_ value of 26.0 μM. Eugenol also inhibited leukotriene C_4_ (LTC4) formation in a concentration-dependent manner with an IC_50_ value of 30.0 μM. Furthermore, the study concluded that the inhibitory effect of eugenol on the 5-LOX enzyme was of a non-competitive nature [54]. Naidu [55] demonstrated that eugenol inhibited 5-LOX-catalysed lipid peroxidation and the inhibitory effect was found to be concentration-dependent. A concentration of 380 μM caused a 50% decrease in lipid peroxidation (IC_50_). Eugenol (33 mg/kg) administered orally for 26 days to rats with severe chronic adjuvant-induced arthritis induced a significant suppression of both paw and joint swelling suggesting that eugenol has potent anti-inflammatory properties [56]. 

### 4.3. Analgesic Activity

Eugenol is a routine analgesic agent widely used in dental clinics due to its ability to alleviate tooth pain. Studies were carried out to evaluate the antinociceptive capacity of eugenol in different experimental pain models in mice including formalin-induced hyperalgesia, acetic acid-induced abdominal constrictions and thermal pain (hot-plate test). A significant inhibition of acetic acid-induced abdominal constrictions was recorded with the maximal effect (92.73% inhibition) at 100 mg/kg. In the formalin-induced paw-licking pain model, eugenol showed evidence of a more pronounced antinociceptive effect in the inflammatory phase (70.33%) than the neurogenic phase (42.22%) at 100 mg/kg. In the thermal pain experiment (hot-plate), a mild reduction in the pain response latency at a dose of 100 mg/kg was recorded [57]. Daniel *et al.* [49] investigated the antinociceptive activity of eugenol *in vivo* (mice) using the acetic acid-induced abdominal writhing method. Eugenol at doses of 50, 75 and 100 mg/kg had a significant antinociceptive effect, compared to the control animals [49]. The antinociceptive effects of orally administered eugenol in concentrations of 1–10 mg/kg were examined in ICR mice. Eugenol exhibited an antinociceptive effect in a dose-dependent manner as measured by the number of contractions of the body and this activity could be maintained for at least 30 min [58].

The transient receptor potential vanilloid 1 (TRPV1) receptors are involved in the transmission and modulation of pain, as well as the integration of diverse painful stimuli. As a vanilloid compound, it is expected that eugenol activates TRPV1 channels in the peripheral nervous system. The pain sensation is augmented by the acidic extracellular pH, and Ca^2+^ influx through activated voltage-or ligand-gated cation channels has been recognised to lower the intracellular pH in neurons [59]. A study was conducted in order to determine the effects of eugenol on the inhibition of TRPV1 and the results showed that eugenol inhibited voltage-activated Na^+^ and Ca^2+^ channels [60,61]. In the central nervous system (CNS), eugenol enhanced spontaneous excitatory transmission in the substantia gelatinosa (SG) in rat spinal cord slices, producing an outward current at −70 mV due to a large increase in the frequency of spontaneous excitatory postsynaptic current (sEPSC). The concentration-dependent effect of eugenol (EC_50_ = 3.8 mM) on sEPSC was not affected by capsazepine (TRPV1 antagonist) but inhibited by ruthenium red (nonspecific TRP antagonist) and HC-030031 (TRPA1 antagonist). The mechanism of action was deduced to be the activation of TRPA1 channels leading to an increase in the spontaneous release of l-glutamate to SG neurons. A membrane hyperpolarisation not mediated by TRP channels was also produced [61]. In another study, Lee *et al.* [62] investigated the effect of eugenol on the high-voltage-activated Ca^2+^ channel (HVACC) currents in dental primary afferent neurons. They reported that eugenol inhibited HVACC currents in both capsaicin-sensitive and capsaicin-insensitive dental primary afferent neurons, suggesting that the HVACC inhibition by eugenol might contribute to its analgesic effect.

In a monoiodoacetate-induced rat model (*n* = 6/group) of osteoarthritis, eugenol showed positive analgesic effects. Daily administration of eugenol (40 mg/kg) by gavage starting 2 days after osteoarthritis induction resulted in significant positive changes in dynamic gait parameters of the affected limb. In addition, spinal cord pain-related peptides substance P and calcitonin gene-related peptide (CGRP) levels decreased while dynorphin increased [63].

The anaesthetic capacity of eugenol was evaluated in juvenile *Leporinus macrocephalus,* a fish species also known as “piavuçu”. Eugenol had strong anaesthetic effect on *L. macrocephalus* and a dose of 37.5 mg/L of eugenol was recommended for the fast and safe anesthesia of piavuçu juveniles (*n* = 72) [64]. In a similar investigation, the efficacy of benzocaine and eugenol as anaesthetics in *Trachinotus marginatus* juvenile fish was studied. Benzocaine and eugenol, at 50 ppm, induced fast anaesthesia and recovery (3 min and 5 min, respectively) [65]. In European sea bass (*Dicentrarchus labrax*) (*n* = 240), eugenol induced deep anaesthesia without substantially affecting their blood profile as measured by serum cortisol, glucose and haematocrit values. Induction was rapid but recovery was very slow even at low concentrations [66]. Eugenol (20 µL/L) was investigated in sub-adult and post-larvae white shrimp (*Litopenaeus vannamei*) and determined to be effective at inducing anaesthesia after 6 h [67].

### 4.4. Anti-Oxidant Activity

There is a growing tendency to replace synthetic anti-oxidants with natural compounds. Many phenolic essential oil constituents (e.g., carvacrol, thymol) have demonstrated their capacity as anti-oxidant molecules. The anti-oxidant activity of eugenol and its isomer isoeugenol was investigated using iron-mediated lipid peroxidation and auto-oxidation of Fe^2+^. The inhibitory effect of eugenol on lipid peroxidation was less potent with an IC_50_ value of ≈80 μM, which was eight-fold the value of isoeugenol [68]. The mechanism of action of the two compounds was investigated and it was suggested that the anti-oxidant capacity of eugenol could be explained by the formation of complexes with reduced metals. The potent inhibitory effect of isoeugenol on lipid peroxidation may be related to the decreased formation of the perferryl ion or iron-oxygen chelate complex as the initiating factor of lipid peroxidation [68]. Another study found that eugenol dose-dependently exhibited anti-oxidant capacity using the hydroxyl radical scavenging and 2,2-diphenyl-1-picrylhydrazyl (DPPH) tests. At a volume ranging from 5 to 25 μL, the percentage inhibition ranged from 41 to 93% and from 39 to 62% against the DPPH and hydroxyl radicals, respectively [16]. Twenty essential oil constituents from various groups (alcohols, aldehydes, ketones, esters phenols, *etc.*) were investigated against the DPPH radical. Eugenol was the most active compound, with an IC_50_ value of 46.6 μM [19]. In general, at low concentrations eugenol has an anti-oxidant and anti-inflammatory effects, whereas at high concentrations it acts as a pro-oxidant, leading to tissue damage resulting from the enhanced generation of free radicals [69]. Clove oil showed promising anti-oxidant activity with an IC_50_ value of 8.85 μg/mL against the DPPH radical [48]. 

Superoxide and hydroxyl radicals have been implicated in Parkinson’s disease (PD) pathogenesis, leading to the increased investigation on the use of free radical scavengers and anti-oxidants as a potential strategy to slow disease progression. PD was induced in rats through the injection of 6-hydroxydopamine (6-OHDA) which reduced the dopamine level in the striatum, and the rats were treated with L-DOPA and eugenol. The eugenol enhanced the dopamine depression although a previous report indicated that pre-treatment with eugenol prevented 6-OHDA-induced dopamine depression. Therefore, it is important to note that although pre-treatment may be beneficial, using eugenol after the onset of PD symptoms may be deleterious [70].

### 4.5. Anticancer Activity

Due to the toxic effects of synthetic drugs, natural agents that can inhibit, delay, block, or reverse the initiation and promotional events associated with carcinogenesis is needed for the prevention and/or treatment of cancer. Several essential oil constituents, including eugenol and its related compounds, have been investigated for anticancer activity. Eugenol (500 μM) was found to reduce cell viability of HeLa cells [71]. Eugenol was tested alone and in combination with a chemotherapeutic drug (gemcitabine) to evaluate its inhibitory effect against cancer cells. Eugenol showed dose-dependent selective cytotoxicity towards HeLa cells in comparison to normal cells. Furthermore, eugenol and gemcitabine in combination induced growth inhibition and apoptosis (programmed cell death) at lower concentrations in comparison to the individual compounds indicating synergistic interactions [71]. Eugenol (150 μM) and gemcitabine (15 μM) resulted in a decrease in cell viability from 84% (eugenol alone) and 51% (gemcitabine alone) to 47% in combination. Treatment of colon cancer cells with eugenol resulted in the reduction of intracellular non-protein thiols and an increase in the earlier lipid layer break. In addition, dissipation of mitochondrial membrane potential and generation of reactive oxygen species accompanied the eugenol-induced apoptosis [72].

In order to determine the mechanism of action of eugenol against the HL-60 cell line, the induction of DNA fragmentation was demonstrated by incubating HL-60 cells with eugenol at various concentrations and time intervals. A gradual increase of fragmented DNA could be observed. A ladder pattern of internucleosomal DNA fragmentation was also apparent when cells were treated for 4 h with 40 μM of eugenol. However, the fragmentation of DNA was totally inhibited by pretreatment with the anti-oxidant N-acetyl-L-cysteine. This indicated that eugenol induced apoptosis in HL-60 cells via reactive oxygen species generation, by inducing mitochondrial permeability transition, by reducing anti-apoptotic protein bcl-2 level and cytochrome *c* release to the cytosol as well as subsequent apoptosis [73].

The effect of eugenol and its isomer isoeugenol to inhibit the proliferation of melanoma cells was investigated in a B16 xenograft model. The results indicated that eugenol was a potent inhibitor of melanoma cell proliferation producing a significant tumour growth delay, approximately 40% decrease in tumour size, and a 19% increase in the median time to end point. Furthermore, about 50% of the animals in the control group died as a result of metastatic growth, whereas none in the treatment group showed any signs of invasion or metastasis. The antiproliferative mechanism of eugenol was further investigated in the human malignant melanoma cell line (WM1205Lu) which showed that eugenol arrests cells in the S phase of the cell cycle and induces apoptosis [74]. 

In another attempt to understand the mechanism of action of essential oil constituents, the *in vitro *antiproliferative effects of eugenol and its isomer isoeugenol on cell cycle progression of human epidermoid carcinoma A431 cells was investigated. The two compounds exhibited an antiproliferative effect by blocking the cells in the G0/G1 phase of the cell cycle. Both compounds favoured the translocation of the aryl hydrocarbon receptor into the nucleus, aryl hydrocarbon receptor target gene expression and aryl hydrocarbon receptor-dependent modulation of cell cycle regulatory proteins [75]. 

Several studies have shown that a combination of substances may produce better activity than when a single component is used and combination therapy is often used for diseases such as malaria, HIV and cancer. A combination of eugenol and 2-methoxyestradiol, an endogenous estrogenic metabolite reported to be an antiproliferative agent in various tumour models, inhibited the growth of prostate cancer cells and induced apoptosis in a synergistic manner [76]. 

Some researchers pointed out the effect of functional groups on the biological activity of certain molecules. In order to evaluate the effect of an hydroxyl group on biological activity, the anticancer activity of eugenol and its analogues were investigated *in vitro* on two human cancer cell lines namely androgen-insensitive prostate cancer cells (DU-145) and oral squamous carcinoma cells (KB) using the cell viability tetrazolium salt assay. Eugenol exhibited activity with IC_50_ values of 30.39 × 10^6^ and 28.48 × 10^−6^ DU-145 and KB cells, respectively. The eugenol analogues 5-allyl-3-nitrobenzene-1,2-diol and 4-allyl-2-methoxy-5-nitrophenyl acetate were found to be more active than eugenol with IC_50_values in DU-145 cells of 19.02 × 10^−6^ and 21.5 × 10^−6^ mol/L, respectively, and in KB cells of 18.11 × 10^−6^ and 21.26 × 10^−6^ mol/L, respectively. The results obtained demonstrated that the nitro and hydroxyl groups could play an important role in the activity of these compounds [77]. The anticancer activity of eugenol and methyleugenol was evaluated on the HL-60 human promyelocytic leukemia, U-937 human histocytic lymphoma, HepG2 human hepatoma, 3LL Lewis mouse lung carcinoma and SNU-C5 human colon cancer cell lines using a colourimetric assay (MTT). Eugenol showed varying degrees of toxicity with IC_50_ values ranging from 23.7 to 129.4 μM. Chemical transformation of eugenol to methyleugenol in order to elucidate the role of the OH-group on the biological effect showed that the activity of methyleugenol was weaker with IC_50_ values ranging from 89.3 to 300 μM [73]. 

### 4.6. Antimutagenic and Antigenotoxic Activities

Since the twentieth century, attention has been devoted to the antimutagenic and genotoxic effects of natural compounds. The possible reduction of the mutagenicity and genotoxicity of benzo[α]pyrene (B[α]P) by eugenol was tested *in vivo* on the *k-lacZ-transgenic *mouse strain 40.6 (MutaT~Mouse). No evidence of mutagenicity or genotoxicity was noted but the eugenol diet (0.4% w/w) resulted in apparent growth retardation in comparison with the control group, although the differences were not statistically significant [78]. 

Eugenol was investigated *in vitro* in order to evaluate its antigenotoxic effects in unscheduled DNA synthesis with established mutagens and the *Salmonella typhimurium *mutagenicity assays. In addition, the effect of *in vitro *treatment with eugenol on benzo[α]pyrene (B[α]P)-induced genotoxicity in the human hepatoma cell line Hep G2 was determined. The mutagenicity of B[α]P in the *S. typhimurium *mutagenicity assay was lower in liver S-9 fractions prepared from rats treated orally with eugenol (1,000 mg/kg body weight) than in liver S-9 fractions from control rats, while in the unscheduled DNA synthesis assay, eugenol showed no effect. *In vitro *treatment of cultured cells with eugenol resulted in an increase in genotoxicity of benzo[α]pyrene suggesting that the antigenotoxicity of eugenol *in vivo* is rather limited [79]. 

Ramos *et al.* [80] investigated the oxidative mutagenesis induced by *tert*-butylhydroperoxide (TBH) in *E. coli *IC 188 and found that when eugenol was added to TBH, at 300–400 μg/plate, a significant decrease of 50% in the oxidative mutagenesis by TBH was recorded. In the mouse bone marrow micronucleus test, pretreatment with *trans*-anethole and eugenol resulted in significant antigenotoxic effects against cyclophosphamide procarbazine, N-methyl-N′-nitro-N-nitrosoguanidine and urethane [81]. Sukumaran and Kuttan [82] studied the effect of eugenol on tobacco-induced mutagenesis using the Ames Salmonella/microsome assay. The results indicated that eugenol inhibited tobacco-induced mutagenicity at concentrations of 0.5 and 1 mg/plate. However, a study on humans showed contrasting results. No significant differences on the cytogenetic parameters were noted after ten healthy non-smoking males ingested 150 mg of eugenol per day suggesting that eugenol has no antigenotoxic potential in humans [83]. 

### 4.7. Modulatory Effects

Several studies have demonstrated that oxygen free radicals formed by xanthine/xanthine oxidase (X/XO) may be involved in the *N*-methyl-D-aspartate (NMDA)-mediated neurotoxicity and inhibitory action of glutamate uptake in glial cells [84,85,86]. The neuroprotective efficacy of eugenol against *N*-methyl-d-aspartate-induced neurotoxicity, oxygen-glucose deprivation-induced neurotoxicity and xanthine/xanthine oxidase-induced neurotoxicity in primary murine cortical cultures was investigated. The results showed that eugenol at concentrations of 100–300 mM attenuated NMDA induced acute neurotoxicity by 20–60%. Similarly, eugenol (300 mM) also inhibited NMDA-induced elevation in neuronal 45 Ca^2+^ uptake by 10–30%. Furthermore, it was observed that addition of eugenol (100–300 mM) prevented acute neuronal swelling and reduced neuronal death by 45–60% in a concentration-dependent manner and oxidative neuronal injury induced by X/XO was also significantly reduced (75–90%) [87].

Mahapatra *et al.* [88] investigated the *in vitro* protective effect of eugenol (1–20 μg/mL) against nicotine-induced (10 mM nicotine) cellular damage in mice peritoneal macrophages by analysing the radical generation, lipid, protein, DNA damage and endogenous anti-oxidant status. The results indicated that eugenol could be used as modulator of nicotine-induced cellular damage and immunomodulatory drug against nicotine toxicity. A significant increase in the level of radical generation, NADPH oxidase and myeloperoxidase activity lipid, protein, DNA damage and oxidised glutathione level was noted in the nicotine-treated group and significantly reduced by addition of eugenol. Furthermore, the anti-oxidant condition was significantly depleted in the nicotine-treated group, which was effectively restored by addition of eugenol [88].

## 5. Other Pharmacological Properties

Eugenol was investigated for its anti-ulcerogenic effects. It was found that gastric ulcer formation, induced by administration of two ulcerogenic agents was significantly reduced through pre-treatment with eugenol (10–100 mg/kg, p.o.). Similarly, eugenol also reduced the gravity of lesions [89]. In rats with indomethacin-induced ulcers, pretreatment with eugenol (100 mg/kg, orally) 60 min before indomethacin administration reduced gastric mucosal lesions and gastric acid outputs, resulting in a gastroprotective effect. The possible anti-ulcer mechanisms of actions proposed were the opening of ATP-sensitive potassium (KATP) channels, free radical scavenging, decreased acid-pepsin secretion, increased mucin production and prevention of the deleterious rise in nitric oxide level [90].

The results of an *in vivo* study on rats demonstrated that eugenol reduced diarrhoea induced by castor oil, the tone of isolated gut muscle and myometrium, the rate of intestinal transit and the intestinal accumulation of fluid induced by PGE_2_ [91]. In order to investigate some of the mechanisms involved in the spasmolytic and relaxant effects of eugenol, the effects of eugenol on contractions of isolated rat ileum induced by electrical field stimulation (EFS) were studied. Eugenol (100 μM) significantly and reversibly reduced EFS induced contractions by approximately 80%. Control contractions were 2.0 ± 0.18 g, while contractions in the presence of eugenol decreased to 0.4 ± 0.02 g, respectively [92]. The administration of eugenol also has a myogenic antispasmodic effect on the airway smooth muscle of rats. After EFS in the tracheal muscles, eugenol in concentrations of 1–2,000 µM reduced contractions. This effect was not altered by montelukast or indomethacin suggesting the effects are not modulated by arachidonic acid derivatives. The mechanisms involved seem to include blockade of voltage- and receptor-operated Ca^2+^ channels, IP 3-induced Ca^2+^ release from sarcoplasmic reticulum and reduction of the sensitivity of contractile proteins to Ca^2+^ [93].

Eugenol was intragastrically injected in rabbits to evaluate its potential in reducing fever. Results showed that eugenol exhibited pronounced antipyretic activity when given intravenously and intragastrically and may decrease fever through a central action that is similar to that of allopathic antipyretic drugs such as acetaminophen [94]. 

Monoamine oxidase inhibitors (MAOIs) are drugs used in the treatment of depression. The antidepressant-like activity of eugenol and three selected eugenol analogues was tested in CD-1 (ICR) mice using an established antidepressant screening test (forced swim test). The results indicated that eugenol exhibited anti-depressant like effects against monoamine oxidase type A and type B with the concentration required to produce half maximum inhibition *K*_i_ of 26 and 211 μM, respectively [95]. Stress is believed to be one of the leading psychopathological causes for several mental disorders [96]. Some physiological and psychological responses to stress are mediated by the hypothalamic-pituitary-adrenal (HPA) axis, sympathoadrenal and brain monoaminergic systems. A study was designed to evaluate the anti-stress effect of eugenol in the 4-h restraint rat model. Ulcer index was measured as a parameter of the stress response. The HPA axis and the sympathoadrenal system (SAS) were monitored by estimating plasma corticosterone and norepinephrine (NE), respectively. Analysis of NE, serotonin (5-HT), dopamine, and their metabolites in discrete brain regions was performed to understand the role of brain monoaminergic systems in the anti-stress effect of eugenol. The study showed that stress exposure increased the ulcer index as well as plasma corticosterone and NE levels. Eugenol pretreatment for 7 days decreased the stress-induced increase in ulcer index and plasma corticosterone but not NE levels, indicating a preferential effect on the HPA axis [96]. The mechanisms involved in the ability of eugenol to affect voltage-gated ion currents and neuronal excitability was investigated by Huang *et al.* [97]. Eugenol increased the degree of INa activation and reversibly suppressed non-activating INa. In addition, at higher concentrations eugenol diminished L-type Ca^2+^ current and delayed rectifier K^+^ current. In pilocarpine-induced seizures in rats, a lower seizure severity and mortality was noted, though no shorter seizure latency effect was observed. The mechanism of action was deduced to be the synergistic blocking of INa and non-activating INa affecting neuronal spontaneous action potentials (AP) [97].

In the ovariectomised (OVX) rat model of osteoporosis, the hydroalcoholic extract of dried clove buds rich in phenolic compounds such as eugenol and eugenol derivatives showed favourable effects on bone-preserving efficacy. The induced responses on serum alkaline phosphatase, serum tartrate-resistant acid phosphatase, and urinary calcium, phosphate and creatinine were significantly restored after supplementation with the extract [98]. 

## 6. Agricultural Applications

New potential safe strategies for control of postharvest decay in crops are needed due to the problems related to synthetic fungicides. Postharvest diseases cause heavy losses of fruits during storage and species such as *Phlyctema vagabunda*, *Penicillium expansum*, *Monilia fructigena* and *Botrytis cinerea* are reported to damage apples in many regions of the world. The *in vitro* and*in vivo* activities of two eugenol formulations (eugenol-Tween^®^; eugenol-ethoxylate) against the four apple pathogens revealed growth inhibition of the pathogens incorporated in malt extract agar medium with an MIC value of 2 mg/mL. In addition, the mycelial growth of the four test pathogens was completely inhibited when treated with 150 μL/L of volatile eugenol [99]. Combrinck *et al.* [100] investigated the effects of eugenol on various pathogens causing postharvest decay of fruit. The lowest concentration required to achieve 100% inhibition for *Lasodiplodia theobromae*, *Alternaria citrii*, *Penicillium digitatum* and *B. cinerea *was 500 μL/L.

*Alicyclobacillus acidoterrestris* is a thermo-acidophilic, non-pathogenic, spore-forming bacterium detected in spoiled commercial pasteurised fruit juice. Several fruits such as apple, white grape and tomato are predominantly vulnerable to this pathogen. Studies were conducted to determine the ability of eugenol to control spore germination of *Alicyclobacillus acidoterrestris*. The results indicated that spore germination could be inhibited through the use of 80 ppm of eugenol or alternatively through the combination of 40 ppm of eugenol with 20 ppm of cinnamaldehyde [101]. Inhibition of the wheat seed germination by clove oil was investigated and eugenol was found to be responsible for its strong inhibitory activity [102]. In stored sorghum grains infected with *Aspergillus flavus*, the use of eugenol (8.205 mg/g) showed an antifungal effect with complete inhibition of aflatoxin B1 production [103]. 

The effect of eugenol alone and in combination with cinnamaldehyde against the wood decay fungi, *Lenzites betulina* (white-rot fungus) and *Laetiporus sulphureus* (brown-rot fungus) was evaluated using the MIC method which involved serial dilutions of the compound with sterilised potato dextrose agar (PDA) in Petri dishes containing 15 mL agar. Eugenol exhibited good activity against *L. betulina* (IC_50_ value: 0.37 mM and MIC value: 0.70 mM) and *L. sulphureus* (IC_50_ value: 0.52 mM and MIC value of 0.65 mM) [104]. Synergistic interactions were noted when eugenol and cinnamaldehyde were combined in a 1:1 ratio with MIC values of 0.68 and 0.40 mM against *L. betulina* and *L. sulphureus*, respectively. This synergistic effect was attributed to the interference in fungal cell wall synthesis and cell wall destruction in addition to a radical scavenging effect. The combination of eugenol (0.5 mg/mL) and thymol (0.125 mg/mL) was found to induce a significant increase in the number of cells damaged in comparison to the corresponding single concentration of the two molecules after an incubation period of 4 h [104].

## 7. Insecticidal and Fumigant Properties

In recent years, natural insecticides have been developed due to the global concern about air pollution through the use of synthetic insecticides. Crude essential oils and some of their constituents have been identified as a source of natural pesticides. The repellent effects and fumigant potency of *Ocimum gratissimum* oil (≈64% of methyleugenol) and eugenol were evaluated against the rice weevil (*Sitophilus oryzae*), one of the most severe stored-grain pests worldwide, the Rust Red Flour Beetle (*Tribolium castaneum*), the saw-toothed grain beetle (*Oryzaephilus surinamensis*), the lesser grain borer (*Rhyzopertha dominica*) and the Chinese bean weevil (*Callosobruchus chinensis*). The fumigant toxicity of the oil and eugenol were assessed at various concentrations (0, 1, 5 and 10 mL/L air) in space fumigation, whereas repellency was evaluated at doses of 0, 1.0, 2.0, 3.0 and 4.0 mL oil/2 g grain [7]. The results showed that fumigant toxicity and repellency of the oil and eugenol were significantly influenced by concentration and time after treatment. The oil was found to be more active than eugenol alone. At 1 mL/L air, the oil caused 98%, 99% and 100% mortality of *R. dominica*, *O. surinamensis* and *C. chinensis*, respectively, 24 h after treatment, whereas eugenol alone could only achieve 79%, 61% and 100% mortality. Percentage repellency values ranged from 37.5% to 100% and 45% to 100% for the oil and eugenol, respectively, against all the insects tested [7]. 

The toxicity and protectant potential of eugenol against four Coleoptera (beetle) species (*Sitophilus granarius*, *Sitophilus zeamais*, *Tribolium castaneum* and *Prostephanus truncalus*) of stored products was investigated. Eugenol applied topically impregnated on filter papers, whole grains or glass pebbles was highly toxic to all four species. Furthermore, eugenol was highly repellent to the four beetle species tested with overall repellency in the range of 80–100%. Eugenol also inhibited the development of eggs and immature stages inside grain kernels [105]. 

In another study, the toxicity of eugenol and related compounds (isoeugenol and methyleugenol) used alone and in combination with the pyrethroid insecticide deltamethrin, and the effects of these benzene derivatives on insect nutrition was investigated against maize weevil (*Sitophilus zeamais*) and the red flour beetle (*Tribolium castaneum*). The three oil components exhibited comparable contact toxicity to *S. zeamais* (LD_50_ values ≈ 30 mg/mg insect). Different levels of activity were noted against *T. castaneum* with isoeugenol exhibiting the greatest activity and methyleugenol the lowest activity. It was also found that eugenol significantly reduced food consumption in adult *S. zeamais* at a concentration of 13.2 mg/g [106].

Eugenol was tested for its effect on total microbial count of bacteria, yeasts and moulds in air and was found to reduce the total microbial count. Eugenol reduced the total microbial count of bacteria by 69.4% and yeasts and moulds by 58.3% [107]. The fumigant toxicity of various essential oil constituents were investigated against acaricide-susceptible, chlorfenapyr-resistant, fenpropathrin-resistant, pyridaben-resistant and abamectin-resistant strains of female red spider mites (*Tetranychus urticae* Koch). The results indicated that eugenol was highly toxic with an LC_50_ value of 24.6 μg/cm^3^. The fumigant toxicity of eugenol was almost similar against females from either of the susceptible and resistant strains, indicating that eugenol and acaricides do not share a common mode of action or elicit cross-resistance [108].

## 8. Skin Permeation Enhancement

The complicated architecture and composition of the stratum corneum limits the number of molecules that can be transported passively across the skin. Transdermal drug delivery is a common means to increase drug permeation and the delivery of drugs across human skin has gained a wide acceptance as a viable administration route for potent and low molecular weight therapeutic agents. Many approaches have been investigated to overcome skin impermeability such as the use of chemical permeation enhancers and iontophoresis which is the use of a small amount of electric charge to deliver a chemical substance through the skin. Several essential oils components (e.g., linalool, menthol and limonene) have been investigated as permeation enhancers and have shown promising results [109,110]. The effect of eugenol as permeation enhancer (in combination with 50% ethanol) was investigated in the percutaneous absorption of tamoxifen, an antagonist of the estrogen receptor in breast tissue. It was found that eugenol significantly enhanced the permeability of tamoxifen through porcine epidermis in comparison with the control (50% ethanol) [111]. The effect of clove oil on the transdermal delivery of ibuprofen was evaluated in rabbits using both *in vitro* and *in vivo* experiments. The *in vitro* results indicated a significant permeation enhancement effect of the oil with an enhancement ratio of 7.3. The flux value of the 1% oil application group was 239 μg/cm^2^/h, while the 3% oil group had a flux value of 293 μg/cm^2^/h. Although the *in vivo* results also demonstrated a significant permeation enhancement effect, this enhancement was lower compared to the *in vitro* experiment. The 3% oil application group had an area under the curve value of 80.8 μg/mL.h which was 2.4 times higher than the control. GC-MS results indicated the two major compounds to be eugenol and acetyleugenol (90.93% of the total oil), therefore it was suggested that these constituents contribute to the permeation enhancing ability [112]. 

## 9. Toxicity and Allergenicity of Eugenol

Eugenol has been classified as ‘generally recognised as safe (GRAS)’ by the U.S. Food and Drug Administration [113]. However, it is necessary to determine the toxicity of eugenol as higher concentrations are used for other applications. In addition, chronic exposure remains to be investigated. Okada *et al.* [114] examined the cytotoxicity and internucleosomal DNA fragmentation of eugenol and related compounds (2-methoxy-4-methylphenol, 3,3′-dimethoxy-5,5′-di-2-propenyl-1,1′-biphenyl-2,2′-diol and 3,3′-dimethoxy-5,5′-dimethyl-1,1′-biphenyl-2,2′-diol) using leukemia cells (HL-60). The IC_50_ values obtained ranged from 0.18 to 0.38 mM and DNA fragmentation was induced most strongly by 3,3′-dimethoxy-5,5′-dimethyl-1,1′-biphenyl-2,2′-diol followed by eugenol. The DNA-damaging effects and cytotoxicity of eugenol and borneol were investigated in malignant HepG2 hepatoma cells, malignant Caco-2 colon cells, and nonmalignant human VH10 fibroblasts using the trypan-blue exclusion assay. The results demonstrated that the cytotoxicity of eugenol against the three cell lines was significantly higher than that of borneol. Furthermore, it was observed that borneol did not cause any DNA strand-breaks at the concentrations studied, but that eugenol at concentrations lower than 600 μM significantly increased the level of DNA breaks in human VH10 fibroblasts and to a lower degree in Caco-2 colon cells [115]. The *in vitro* toxicity of this compound on transformed kidney epithelial cells was low (IC_50_ value: 1358 μM), approximately 20 times lower toxicity than quinine [19]. 

The genotoxicity of eugenol, isoeugenol and safrole was investigated in the wing spot test of *Drosophila melanogaster* (common fruit-fly) using the *Drosophila *wing somatic mutation and recombination tests. Eugenol and safrole produced a positive recombinagenic response which was related to a high CYP450-dependent activation capacity. It was suggested that the reactive metabolites of eugenol and recombinagenic compounds were responsible for the genotoxicity of eugenol [116]. The genotoxicity of eugenol in V79 cells using chromosomal aberrations (CAs), with and without rat liver biotransformation (S9), was investigated *in vitro.* Eugenol was found to induce CAs, with significant increases (3.5% aberrant cells) at 2,500 μM, demonstrating cytotoxicity at higher doses. Furthermore, S9 increased the induction of CAs in a dose-dependent manner to 15% at 2,500 μM, with a high frequency of chromatid exchanges [117]. 

The acute toxicity of eugenol was studied *in vivo* by exposing three groups of ten Sprague-Dawley rats (five male; five female) to three different concentrations of eugenol (2.58, 1.37 and 0.77 mg/L) delivered as a submicron aerosol for 4 h followed by 14 days of observation. No mortality was noted during the study period and clinical signs observed included moderate increased salivation, agitation indicative of irritation and abnormal breathing patterns. The clinical symptoms resolved itself during the 1st day. Marked reductions in food (80%) and water (40–70%) intake overnight following exposure were recorded and body weight decreased as well [118]. Studies were carried out to determine whether the use of eugenol and its isomer isoeugenol have sensitisation effects using the murine local lymph node assay and the guinea pig maximisation test. It was found that neither eugenol nor isoeugenol have a significant potential to cause sensitisation of the respiratory tract [119]. However, intravenous infusion of eugenol in rats at 4 μL and 8 μL (6.52 mol/L) caused acute respiratory distress with haemorrhagic pulmonary oedema [120]. 

Several adverse effects related to the use of dental products containing eugenol have been observed and include localised irritation of the skin, ulcer formation, allergic contact dermatitis, tissue necrosis, reduced healing and in rare cases even anaphylactic-like shock [121]. Severe side-effects after clove oil ingestion were reported by Hartnoll *et al.* [4]. A two-year old boy ingested between 5 and 10 mL of clove oil and on arrival at the hospital one hour later presented as normal but slightly drowsy, distressed and crying. Within 3 h his condition deteriorated into deep coma with marked acidosis, within 8 h, his blood glucose concentration was undetectable and he suffered a generalised seizure, and within 24 h deteriorating liver function as well as disseminated intravascular coagulopathy (DIC) was noted. After intensive symptomatic treatment the patient regained consciousness on the 6th day after ingestion and eventually made a full recovery. There are several similarities between eugenol and paracetamol poisoning in terms of its hepatotoxic effects [4]. Investigation into the metabolism of eugenol in eight healthy human volunteers (four males and four females) after oral administration of 150 mg of eugenol in gelatine capsules revealed that eugenol is absorbed quickly (after 24 h). More than 55% of the dose administered is excreted in the urine after metabolism in the liver as glucuronic acid or the sulfate conjugate of eugenol. *Cis* and *trans*- isoeugenol, 3-(4-hydroxy-3-methoxyphenyl) propane, 3-(4-hydroxy-3-methoxyphenyl) propionic acid and eugenol epoxide were among the conjugated metabolites identified [122]. Thompson *et al.* [123] found that eugenol conjugated with sulfate, glucuronic acid and glutathione with glucuronic acid being the major conjugate in an *in vitro* study using isolated rats hepatocytes. This study showed hepatotoxicity with cell death occurring in >85% of rat hepatocytes 5 h after exposure. In the case of an overdose alternative metabolic pathways are utilised due to saturation, a process supported by acetylcysteine administration and cell death in rat hepatocytes was prevented completely by co-administration [4,122].

It is generally accepted that fragrance ingredients are a frequent cause of allergic contact dermatitis [124,125]. The European Union (EU) project SENS-IT-IV addresses the need of developing predictive *in vitro* tests to assess contact and respiratory hypersensitivity reactions [126]. Eugenol is a primary irritant and sensitiser and can cause contact dermatitis as well as irritation of the skin, eyes and respiratory tract. This compound is among the most frequently reported and well-recognised consumer allergens in the European Union [119]. The potential of eugenol and clove leaf oil to induce delayed skin hypersensitivity or to elicit reactions due to pre-existing skin sensitisation in man was evaluated. Analysis of patch-test data demonstrated that eugenol alone or clove oil has a very low potential to cause these effects [127]. In another study, eugenol was found to induce allergic contact dermatitis in guinea pigs [128]. A study on fragrance as an occupational allergen was conducted on a total of 24,046 patients by Buckley *et al.* [124]. For eugenol they found that 14 of 55 (25.5%) health care workers, 145 of 879 (16.5%) non-health care workers, 11 of 29 (39.39%) metalworkers and 148 of 906 (16.3%) people in other occupations were allergic to eugenol. Eugenol is found in soaps, antiseptic solutions and emollient creams used by healthcare workers who frequently wash their hands, dentists are exposed to eugenol in mouthwashes, dressings, impression materials and periodontal packings, eugenol is present in 27% of household products, it is commonly included in cutting fluids used by metalworkers and massage therapists are frequently exposed due to the use of essential oils in their trade [124]. Recently Svedman *et al.* [129] evaluated the potential of eugenol to cause allergic contact dermatitis in a repeat open application test (ROAT) for a leave-on product. Five volunteers previously sensitised to eugenol were included in the ROAT study where the maximum allowed concentration of eugenol was applied for 4 weeks. Four of the five volunteers reacted during this time, confirming the ability of eugenol to cause contact dermatitis [129]. Due to the allergicity of eugenol, research is now focused on removing eugenol from natural oils and extracts used in perfumery and cosmetics. Bouhlel *et al.* [130] used horseradish peroxidase (HRP) to remove eugenol from rose essential oil without loss of the organoleptic quality and chemical composition. 

## 10. Conclusions

The tropical evergreen clove tree was described by Rumphius as “the most beautiful, the most elegant, and the most precious of all known trees”. It could certainly be described as financially precious during the height of war and competition for control of the valuable clove, when the Portugese chronicler Barros remarked that clove was “the apple of all discord and one could curse it more than gold itself” [2]. Its usefulness did not decline with the tumultuous fluctuations in market value. Research on the properties of clove and its major constituent eugenol has and is still continuing unabatedly with more than 20 articles already published in 2012 as seen in a Scopus^®^ search conducted in April based on the appearance of eugenol in the title. The high number and wide selection of papers attests to its versatility. In structure eugenol is very simple, but it is such a multipurpose molecule with an extensive array of applications in the pharmaceutical, flavour and fragrance, agricultural and other industries. The research avenues have by no means been exhausted and new applications are studied with high frequency. A difference in concentration may lead to a difference in effect such as having either an analgesic or an anaesthetic effect. In addition, small changes in structure using eugenol as starting molecule may have a profound effect on biological activity. For example, it was found that eugenol analogues were more effective (anticancer) due to the presence of hydroxyl and nitryl groups. From several studies, it is evident that synergistic effects are being investigated more frequently. The combination of eugenol and cinnamaldehyde/cinnamate has proved to be particularly effective as antimicrobial and to preserve food products and wood. The mechanism of action of eugenol on micro-organisms positions it as a valuable tool in the fight against infections. In bacteria, damage of the membrane by eugenol caused increased penetration of concomitantly administered antibiotics or other essential oil components. In fungi, eugenol has an effect on envelope morphology and interferes with adhesiveness and transition to the hyphal form thereby preventing colonisation and it also causes cell cycle arrest. It is evident that there is scientific rationale in using eugenol as a functional ingredient to combat infection. 

In stark contrast to many other molecules and plant products, the toxicity of eugenol and clove oil has been fairly well studied though chronic toxicity studies is lacking. It is considered “generally recognised as safe” (GRAS) in food products but accidental ingestion of high quantities of clove oil may have grave consequences. It is worrying that evidence of chromosomal aberrations were found after exposure to eugenol and if we consider its effective fumigant properties, it is apparent that intense toxicity studies should be undertaken to ensure the safety of the general public. The allerginicity of eugenol has received much attention and more recent research is aimed at removing eugenol from oils used as fragrances although its use in household products is unlikely to change. The scent of eugenol has played an imperative role in its applications, but its distinctive odour and its negative connotations have also been studied. Eugenol has been and will remain a mainstay product in restorative dentistry and the emotions evoked by the presence of eugenol in the dentist’s offices have been studied. A neurovegetative analysis revealed that odours form a strong connection with memories and that individual experiences dictated whether a positive or negative effect was induced upon smelling eugenol. It was shown that fear, anger, anxiety and stress could be induced in fearful dental patients through exposure to eugenol [131]. In addition to the well-studied pharmacological properties described here such as antimicrobial, anti-inflammatory, analgesic, anti-oxidant and anticancer activity, other identified activities such as its anti-ulcerogenic potential and effect on osteoporosis and especially its effect on the central nervous system (CNS) encompassing seizure control, Parkinson’s disease, antidepressant effects *etc*. should be further explored. Its abundant potential applications described in this review are sure to be incorporated in future into commercially available products and new uses/processes are lying in wait to be explored.
